# The Influence of Social Media Messaging on Human Papillomavirus Vaccine Attitudes and Confidence Among Adolescent Males: Group Discussion Study

**DOI:** 10.2196/82210

**Published:** 2026-06-03

**Authors:** Neha Trivedi, Rachel Van Vleet, Czesia Eid, Amelia Burke-Garcia, Amy E Leader

**Affiliations:** 1 NORC at the University of Chicago Washington, DC, DC United States; 2 Thomas Jefferson University Philadephia, PA United States

**Keywords:** adolescent males, HPV vaccination, HPV, human papillomavirus, qualitative research, social media influence

## Abstract

**Background:**

Despite the human papillomavirus (HPV) vaccine being available to males for the past 12 years, adolescent males continue to lag in HPV vaccine uptake due to a variety of factors. With the ubiquitous nature of social media use among this population, further research is needed to improve HPV vaccine confidence among young adolescent males using these platforms.

**Objective:**

Using the Elaboration Likelihood Model (ELM), this study sought to better understand knowledge, attitudes, and beliefs about HPV and HPV vaccination among male adolescents aged 14-17 years and examine how social media messaging influences vaccine perceptions and explore the characteristics of persuasion and trustworthiness of digital content narratives across age and vaccination status.

**Methods:**

The study team recruited 18 adolescent males to participate in a series of online focus groups and interviews stratified by age (14-15 years and 16-17 years) and HPV vaccination status. The study team worked with our in-house national probability survey panel, AmeriSpeak Teen Panel, to recruit for the discussion groups and interviews. All discussions were recorded, transcribed, and coded using NVivo 14 (Lumivero). ELM was used to develop discussion guides, codebook, and analysis. Emergent themes were identified, and the full team participated in double-coding and analysis. Data were analyzed using rigorous thematic analysis to identify patterns across groups.

**Results:**

Knowledge of HPV varied by age and vaccination status. Older, vaccinated adolescents were more likely to understand that HPV affects both genders and is sexually transmitted, whereas younger, unvaccinated adolescents often had little to no prior awareness. Parents—especially mothers—were the primary decision-makers for vaccinated adolescents, with older adolescents more likely to be engaged in that decision. Trust in social media health messages was low overall, with participants relying on perceived source credibility over content format or style. Trusted sources included parents, physicians, and well-known health organizations. Younger adolescents were more influenced by personal anecdotes, whereas older adolescents preferred statistics, facts, and reliable sources.

**Conclusions:**

Our findings highlight the importance of tailoring HPV vaccine messaging by age and developmental stage. Trusted messengers, clear factual content, and platform appropriateness are critical for engaging adolescent males. ELM provides a useful lens for interpreting these findings: younger adolescents were more influenced by peripheral cues, personal stories, or visual appeal, whereas older adolescents engaged in more central processing, evaluating the credibility of the source and the factual content of the message. This underscores the need to match message strategies with adolescents’ cognitive and motivational readiness. Future interventions should consider dual strategies targeting both youth and parents, especially for younger adolescents. As the digital landscape continues to evolve, further research should also examine how generative artificial intelligence (AI) may serve as a trusted source or tool for communicating accurate, engaging health information to adolescent audiences.

## Introduction

### Human Papillomavirus Vaccine Uptake Among Adolescents and Slow Adoption of HPV Vaccination Among US Adolescent Males

Human papillomavirus (HPV) remains the most common sexually transmitted infection in the United States despite vaccine availability to prevent many of the infection’s strains in both young women and men [1]. Recent data from the CDC (Centers for Disease Control and Prevention) indicate that HPV vaccine uptake among US adolescents remains suboptimal, with notable gender disparities. In 2023, approximately 72% of adolescents aged 13-17 years initiated the HPV vaccine series, but only 49% completed it [2]. Although the CDC recommends routine vaccination at ages 11-12 years, initiation and completion rates remain consistently higher among females than males. For instance, from 2011 to 2020, the vaccination rate among males increased from 8% to 36%, whereas among females, it rose from 38% to 49% [3]. Studies suggest factors contributing to higher uptake among females include greater health awareness, proactive health care engagement, and stronger recommendations from health care providers [4]. These findings underscore the need for better understanding of the facilitators and barriers to vaccination to ensure equitable access to HPV prevention measures for adolescent males.

While efforts to vaccinate adolescent females for HPV have made strides in reducing HPV infections among this group, HPV vaccination delay, hesitation, and rejection continue to be prevalent among adolescent males [5]. Research suggests that if vaccination levels among adolescent males aged 14-17 years continue to dwindle, rates of HPV-related diseases will rise, continuing to widen vaccination disparities among this population [6]. Numerous interventions to promote HPV vaccination uptake among females exist [7-9], as well as interventions for parents and providers to promote HPV vaccination among younger males [10,11]; however, additional catch-up opportunities are needed for adolescent males aged 14-17 years. Various factors may contribute to the low uptake of HPV vaccination among adolescent males. For example, some health care providers have expressed reluctance to recommend the vaccine to males, citing perceived low risk and concerns about cost-effectiveness [12]. Parents may also be less aware of the vaccine’s benefits for males due to an overall lack of communication about the HPV virus, therefore perceiving HPV-related diseases as less of a concern for their sons [13]. Furthermore, misconceptions that the vaccine promotes promiscuity and general vaccine hesitancy contribute to lower uptake [13].

### Adolescents’ HPV Vaccine Confidence and Health-Related Decision-Making Capabilities

Adolescents aged 14-17 years are developing greater autonomy and self-efficacy, which shape not only their health-related decision-making but also how they engage with health information on social media. Research shows that better knowledge of vaccine-preventable diseases such as HPV, higher confidence in vaccines, and active involvement in the decision-making process may explain a positive relationship with adolescents’ vaccine uptake [14]. Similarly, adolescent maturity and self-efficacy play a role in HPV vaccination decision-making and overall vaccine confidence [15]. Theoretical models of health behavior suggest a role for self-efficacy (one’s belief in one’s ability to perform a certain behavior) in explaining why individuals do or do not engage in health behaviors, and these models have been applied in the context of HPV vaccination [16]. Social media platforms support self-efficacy by allowing adolescents to seek information independently, observe peer norms, and form attitudes outside of parental or clinical settings [17]. Recognizing that this age group prefers to be involved in decision-making, additional information is needed to understand what factors influence communication and messaging about health-related decision-making and vaccine confidence.

Effective communication strategies, particularly those initiated by health care providers, have been shown to increase vaccine uptake. However, given adolescents’ high engagement with digital platforms, social media now plays a critical role in shaping health beliefs and behaviors. Platforms like TikTok, Instagram, and YouTube serve as both information sources and social influence networks. Through these platforms, digital storytelling has emerged as a promising approach; studies have shown the importance of tailored, evidence-based messaging in promoting HPV vaccination among adolescent males and how the evolving digital landscape can help engage this vulnerable age group [18]. This study will add critical information to understanding adolescent males’ motivation and confidence in vaccination decision-making and the role social media plays in vaccine decision-making.

### Social Media Messaging and Influence on US Adolescent Males

Social media plays a pivotal role in shaping adolescent males’ health decisions, including HPV vaccination knowledge, attitudes, and beliefs. Male adolescents often seek health information on platforms like Instagram, TikTok, and YouTube, where content is frequently shared by peers, influencers, or health care professionals [19]. Studies have shown that social media channels can act as positive conduits of message delivery for adolescents if messages are considered interesting, their privacy is protected, and the source is credible [20].

The credibility of these messages is influenced by factors such as the perceived trustworthiness of the source, message framing, and platform-specific features. A systematic review identified 3 key domains affecting adolescents’ trust in health information on social media: trust in the platform, trust in other users, and trust in the content itself [21]. Adolescents tend to trust content that aligns with their social identity, is presented in an engaging manner, and comes from sources they perceive as authentic or relatable [21]. For example, messages delivered by peers or influencers with whom adolescents identify may be more persuasive than those from traditional health authorities. However, other studies suggest that messages developed and delivered by health care organizations or professionals provide reliable and factual information, providing higher believability and trustworthiness of a social media post [22,23].

The rapid evolution of platforms and the constant influx of new content can create an environment in which adolescents are continuously exposed to health-related messages. Studies have shown that exposure to vaccine misinformation on social media is associated with increased vaccine hesitancy, particularly when individuals rely solely on social media for health information without consulting trusted sources such as health care providers [24]. Additionally, the persuasive power of social media is heightened when messages are tailored to resonate with the audience’s values and concerns. For instance, content that emphasizes peer experiences or addresses specific concerns about HPV vaccination may be more effective in influencing adolescent males’ attitudes and behaviors. Therefore, understanding the factors that contribute to trust and engagement on social media is crucial for developing effective health communication strategies aimed at increasing HPV vaccination confidence among adolescent males. Research has yet to show how message characteristics and believability of social media messages targeting adolescent males impact vaccine confidence.

### Theoretical Framework

This study used a qualitative descriptive design grounded in the Elaboration Likelihood Model (ELM), which is well suited for examining how adolescents perceive, interpret, and make meaning of health-related messaging in real-world contexts and social media. ELM posits that when an individual encounters various forms of communication, they process the communication with varying levels of elaboration ranging from low to high [25]. There are multiple factors that contribute to the elaboration of the messaging, requiring the individual to take 2 different routes, peripheral or central. Engaging in lower elaboration, or the peripheral route, results in the individual having lesser ability to process the message and having little or no interest in the subject. By contrast, engaging in higher elaboration, or the central route, results in higher motivation and ability to think about the message and its topic and to react.

More recently, ELM has been adopted to understand attitudes toward COVID-19 vaccination and intention to obtain the vaccine [26]. One study applied ELM to explore the characteristics of COVID-19 vaccine messages that may appeal to Twitter users, with findings suggesting that content-related messaging played a role in shaping decisions regarding whether to retweet antivaccine messages [27]. ELM has also been previously used to understand HPV vaccine messaging on social media to examine intentions and decision-making among parents. One study found that the central route, represented by information quality, affected parents’ perceptions of HPV severity and susceptibility; the peripheral route, represented by source credibility, influenced their perceptions of HPV severity, HPV susceptibility, and vaccine response efficacy [28]. Another study with young girls found that recommended HPV vaccine messages should include focusing on cancer prevention rather than sexual transmission, routinization of the vaccine, and highlighting risks and costs of getting HPV [29]. Lastly, one study found that among college students, the use of hybrid messages containing both statistical and narrative descriptions of HPV resulted in greater perceived risk of getting HPV than either message type alone [30]. However, there has yet to be a study assessing believability of messaging impacting adolescent males aged 14-17 years in their decision-making to obtain HPV vaccines.

### Study Design and Aims

This is an observational study that used qualitative methods to explore the knowledge, attitudes, and beliefs, along with the believability, persuasion, and influence of social media messaging on adolescent males’ HPV vaccine confidence among those aged 14-17 years. Using the ELM framework [31,32], a dual-process communication and psychological framework, as our foundation, we examined how adolescent males process social media messaging (exploring the characteristics and persuasion of various narratives) and how such messages influence believability and adolescent males’ confidence in HPV vaccine uptake.

The purpose of this study was to better understand the characteristics of social media narratives and their impact on adolescent males’ perceptions about and confidence in receiving the HPV vaccine among those aged 14-17 years. ELM guided our protocol development and analysis, focusing on what this group believes and ways they are persuaded, based on social media influence. The study had two aims: (1) to understand knowledge, attitudes, and beliefs about HPV vaccination among male adolescents aged 14-17 years; and (2) to explore the characteristics and persuasive nature of social media (Facebook, TikTok, Twitch, YouTube, etc) narratives to which adolescent males are exposed and whether messaging plays an active role in impacting vaccine confidence among adolescent males.

## Methods

### Study Overview

A qualitative descriptive approach was selected to capture participants’ perspectives in their own words and to generate findings with direct relevance for public health communication practice. The original study design included conducting 4 online focus groups with adolescent males aged 14-17 years to address the 2 key objectives. Using AmeriSpeak (Nation Opinion Research Center [NORC]’s the nationally representative probability panel of more than 30,000 US households) and AmeriSpeak Teen Panel (NORC’s nationally representative sample of US adolescents aged 13-17 years), a group of male adolescents were recruited into the study [33]. The groups were stratified by age and HPV vaccination status: vaccinated males aged 14-15 years, unvaccinated males aged 16-17 years, vaccinated males aged 16-17 years, and unvaccinated males aged 14-15 years. However, due to recruitment challenges with participants aged 14-15 years, the study design was adapted to include a combination of focus groups and individual interviews. This revised approach maintained stratification by age and vaccination status and ultimately improved recruitment outcomes, particularly for the younger age group, through successful scheduling of individual interviews.

### Study Eligibility and Recruitment

To be eligible for this study, parents must have (1) had a male child or male children aged 14-17 years and (2) not expressed strong antivaccine views to the following three questions: (1) “Overall, how hesitant about childhood shots would you consider yourself to be?”; (2) “To what extent do you agree with the following statement: I believe my son should be vaccinated against HPV?”; and (3) “Have you ever spoken with your son about the HPV vaccine?” Additional parental demographics were also collected, which were not part of the eligibility criteria.

Eligibility for adolescent males included (1) being aged 14-17 years and (2) HPV vaccination status (“Yes, I have received both HPV shots and I am fully vaccinated,” “Yes, I have received one HPV shot but I have not received my second dose yet,” “Yes, but I’m not sure if I received both doses,” and “No”). Additional items such as demographics and social media usage were also captured, not part of eligibility criteria. Recruitment and data collection spanned from October 2024 to January 2025.

The panel invited 464 parents, and 326 consented for their adolescent to be offered the study opportunity. Among the 326 male adolescents aged 14-17 years invited to the recruitment screener survey, 111 adolescents were eligible and assented to be contacted for scheduling into a focus group or interview. To ensure homogeneity within each focus group or interview, we stratified participants based on the adolescents’ age and vaccination status.

### Study Procedures

The research team was composed of investigators with training and experience in public health, health communication, qualitative research methods, and vaccine confidence research. Several team members have prior experience designing and evaluating adolescent-focused health communication interventions, which informed the development of the discussion guide and the selection of social media stimulus materials.

The research team developed the focus group discussion guide using ELM to ask questions about knowledge, attitudes, and beliefs around HPV vaccination and to assess the believability of social media messaging on adolescent males’ decision-making and vaccine confidence through message testing. We examined how adolescent males process social media messaging (exploring the characteristics and persuasion of various narratives) and how such messages influence believability and adolescent males’ confidence in HPV vaccine uptake. The discussion guide (Multimedia Appendix 1) was constructed with specific topics (Textbox 1).

**Discussion group section topics.**

**Section topic**

- Knowledge of the human papillomavirus (HPV) and HPV vaccine
- Attitudes and beliefs toward HPV vaccination
- Barriers and motivators to HPV vaccine uptake
- Social media platform use
- Trust/distrust in messages/sources on social media
- Believability of vaccine messages on social media

We also visually presented 4 social media posts on HPV vaccination for discussion. The research team created a social media HPV vaccine media scan protocol to gather a sample of media on HPV vaccination to which adolescent boys may be exposed or that they may actively seek online. Queries were created based on platforms (eg, TikTok, Reddit, X [formerly known as Twitter], Twitch, Facebook, Instagram), time period (eg, past year), content, narrative format, and messenger. TalkWalker [34] and CrowdTangle [35] (before it was obsolete) were used to pull and analyze media posts. Posts were analyzed and discussed as a team for inclusion based on the factors within the query. A matrix was created based on the query terms, and the research team discussed which posts were available and relevant to the study. We compared posts selected from both individuals and organizations, platforms, and messages, including facts compared with narratives (Multimedia Appendix 2). One member of the study team moderated the discussion, while another member was the notetaker. Each focus group lasted approximately 1 hour, whereas interviews lasted approximately 30 minutes.

### Analysis

Multiple strategies were used to ensure the trustworthiness and rigor of the qualitative findings. Credibility was enhanced through the use of verbatim transcripts, double coding of a subset of interviews and focus groups, and regular analytic debriefings among the research team to discuss emerging themes and resolve discrepancies. Upon completion of the discussion groups and interviews, the transcripts were reviewed, updated by inserting moderator questions for clarity, and deidentified. A codebook was developed using a mix of a priori codes based on the discussion guide along with themes that emerged from each discussion group and interview that were relevant to the research questions [36]. Two independent coders from the research team double-coded 4 transcripts (based on the stratification of the groups by vaccination status and age) using NVivo 14 (Lumivero) [37] to conduct qualitative analysis. Following the first round of coding, discrepancies were discussed and addressed, and the codebook was updated where needed. Data saturation was assessed iteratively during analysis, with no new themes emerging across later focus groups and interviews, suggesting that thematic sufficiency was achieved. In addition to double coding by the research team, regular team debriefings, and the use of a structured codebook helped ensure analytic consistency and credibility of findings. Interrater reliability [38] was calculated in MAXQDA using the Cohen κ metric. Using the 4 transcripts, double coding yielded an interrater reliability of 0.74. Codes were then applied to the remainder of the transcripts. Once coding was complete, we structured the presentation of results by themes.

### Ethical Considerations

This study was reviewed and approved by the NORC at the University of Chicago Institutional Review Board (IRB; protocol number 24-07-1895). NORC operates its own IRB, which is registered with the US Department of Health and Human Services Office for Human Research Protections. For projects that are federally funded, the research protocol and accompanying documents have been approved under the expedited review procedure in accordance with HHS Regulations at 45 CFR 46.110 expedited review categories, by the NORC IRB (IRB00000967), under its Federalwide Assurance Number FWA00000142 (FWA 00000142). NORC’s IRB follows a formal process for reviewing all research projects to assure human participants’ protections and minimize respondent burden.

Due to the sensitive nature of the study topic, parents of eligible adolescents provided consent for their adolescent to participate. Both the parent consent language and the adolescent recruitment screener and assent were offered only in English and were self-administered online by the respondent. Consent and assent materials were provided in English and were self-administered online via the AmeriSpeak and AmeriSpeak Teen Panel. No waivers of consent or assent were requested or granted. All discussions were audio-recorded and transcribed. Transcripts were deidentified before analysis (eg, removal of names and other direct identifiers), with moderator questions inserted for clarity without introducing identifying information. Access to deidentified data was restricted to the research team, and results are reported in aggregate to further reduce reidentification risk. Participants received AmeriPoints in the amount of US $50 for participating in the focus group discussion and US $20 for an interview.

## Results

### Study Flow and Participant Distribution

**Figure 1:**
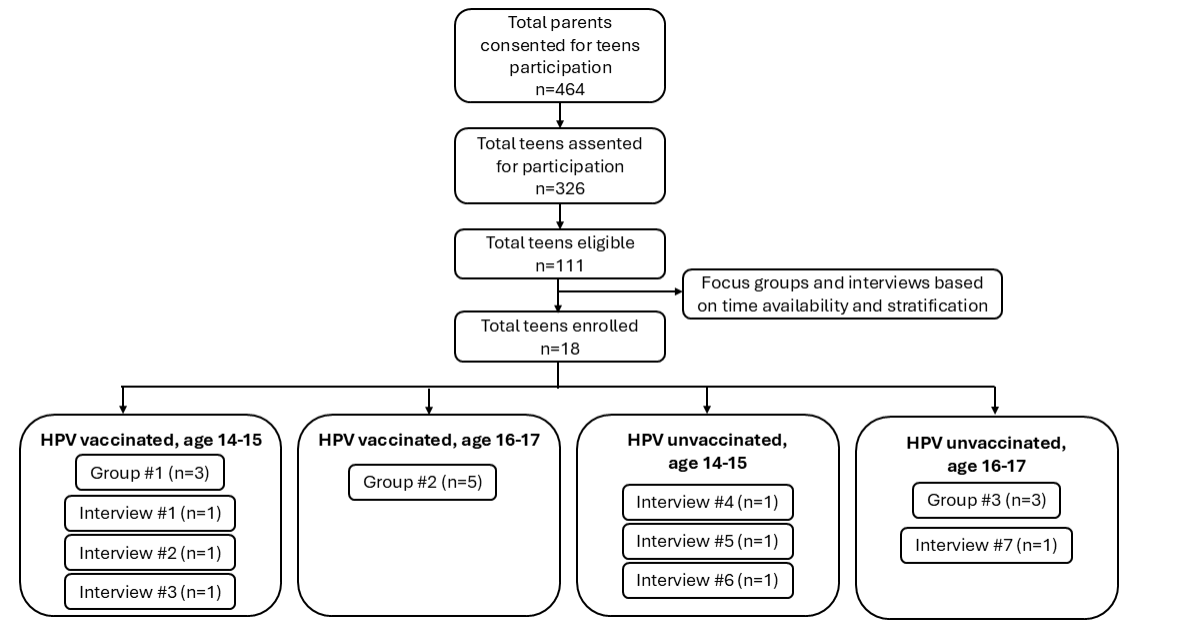
Study flow diagram and recruitment.

Figure 1 provides the study flow and final distribution of participants across data collection methods.

### Sample Characteristics

A total of 18 male adolescents aged 14-17 years participated in either a focus group discussion or an interview.

Table 1 presents the overall characteristics of the adolescent male participants. A total of 9 males were aged between 14-15 years, while 9 males were aged 16-17 years old. Participants were primarily White, non-Hispanic (n=10), and varied in terms of state of residence. The majority (n=11) reported receiving the HPV vaccination, whether 1 dose or both. YouTube (n=15) and Instagram (n=13) were the most used social media platforms.

**Table 1:** Participant characteristics.

Participant characteristics		Frequency, n (%)
**Age (years)**		
	14	5 (28)
	15	4 (22)
	16	3 (17)
	17	6 (33)
**Vaccination status**		
	Yes, I have received both HPV shots, and I am fully vaccinated	8 (44)
	Yes, I have received one HPV shot, but I have not received my second dose yet	0 (0)
	Yes, but I am not sure if I received both doses	3 (17)
	Not vaccinated	7 (39)
**Race/ethnicity**		
	Black, non-Hispanic	3 (17)
	White, non-Hispanic	10 (56)
	Asian-Pacific Islander	1 (6)
	Hispanic	2 (11)
	2+, non-Hispanic	2 (11)
**Household income (US $)**		
	<5000	1 (6)
	15,000-29,999	3 (17)
	30,000-39,999	3 (17)
	40,000-59,999	3 (17)
	85,000-124,999	3 (17)
	125,000-149,999	3 (17)
	175,000-199,999	2 (11)
**State of residence**		
	Arizona	1 (6)
	California	1 (6)
	Florida	3 (17)
	Kansas	1 (6)
	Michigan	1 (6)
	Minnesota	1 (6)
	Missouri	1 (6)
	New York	1 (6)
	North Carolina	1 (6)
	Ohio	1 (6)
	Oregon	1 (6)
	South Carolina	2 (11)
	Tennessee	1 (6)
	Wisconsin	2 (11)
**Social media platform use^a^**		
	Instagram	13 (72)
	Facebook	9 (50)
	Reddit	5 (28)
	Snapchat	11 (61)
	TikTok	10 (56)
	Twitch	5 (28)
	YouTube	15 (83)
	X (formerly known as Twitter)	1 (6)

^a^Respondents reported using multiple social media platforms; total will not equal N=18.

### Thematic Analysis

Findings from this study map clearly onto the core constructs of ELM, which posits that individuals process persuasive information along either the central or peripheral route depending on their motivation and ability to engage with the message. During analysis, transcripts were coded using ELM. ELM codes included central route processing (ability to process, motivation to process, emotional cues, personal relevance, motivation to process, and message quality) and peripheral route processing (familiarity or visual cues, heuristic processing, and source credibility). Social media post text vs source reliability were analyzed to determine how different message features influenced adolescents’ engagement and vaccine-related attitudes. This approach allowed us to explicitly link participants’ reactions to theoretically defined ELM pathways and assess how message characteristics may differentially shape HPV vaccine decision-making.

Table 2 presents results from the thematic analysis and is organized by knowledge, attitudes, and beliefs about HPV and HPV vaccine, barriers and motivators to HPV vaccine uptake, social media use, and trust and believability of vaccine messages on social media. Sample quotes are provided to support findings, based on ELM, and are identified by age and vaccination status of the focus group participant or interviewee (Table 2).

**Table 2:** Thematic analysis of focus group and interview data.

Theme and subtheme (if applicable)	Related ELM^a^ construct	Illustrative quote
Knowledge, attitudes, and beliefs about HPV^b^ and HPV vaccine	Ability to process Motivation to process	I knew boys should get the vaccine from school, so my mom took me to get it, and my aunt had cervical cancer so knew it was something that affects men and women. [FG participant, 16yo, vaccinated]
Barriers and motivators to HPV vaccine uptake	Emotional cues Personal relevance	I’m just cautious of what I put in my body, like any medication. I’m always concerned with side effects. My parents are cautious too. What are the fertility implications? [FG participant, 16yo, unvaccinated]
Social media use	Motivation to process Central route processing Message quality	I don’t really use [social media] for anything research related, I only really use it for talking to my friends or communication because I would rather take some more time using Google or some other website rather than looking on a social media app, because misinformation on there spreads like wildfire and I don’t want to be misinformed about something that I’m trying to learn. So I don’t really use it much for things that are political or medical. [FG, 15yo, vaccinated]
Social media use for health information	Motivation and ability to process Central route processing	We are interested to learn about the risks and the contents of it and eligibility. [FG participant, 17yo, vaccinated] I would like to see more of the health concerns. And maybe the risk, because they usually have that really quick. So people don’t hear about it very much. People need to talk about how you can get it. [FG, 14yo, vaccinated] I’d like it to tell the risks about if you got the vaccine and if you didn’t, like the pros and cons to each. And what is HPV, and more about the risks of that. [IDI, 14yo, unvaccinated]
Trust and believability of vaccine messages on social media	Peripheral route processing (familiarity/visual cues), Heuristic processing Source credibility	It looks relatively believable because it shows a picture of Robert F. Kennedy Jr. The 5thlawsuit he has filed against Merck, challenging the company’s dangerous and defective HPV vaccine for causing severe and life-changing injuries. Now, obviously, in the second picture you can see that he is not in good health. Like he might be smiling there but he does not look happy. Yeah, I’m pretty sure it’s real, but since there is a picture of when he was young and a picture of when he is older sometimes those pictures can be not always right, could be a different person, but I’m pretty sure this is real. [FG, 15yo, vaccinated] Some of these social media messages are old, this one [RFK] is even older, a lot of things can change in a vaccine, modern medicine advances.I don’t know if it’s true, anyone can get hospitalized – that’s a lot of likes but don’t know if that matters that much. [IDI, 14yo, vaccinated] Since Elon bought it [Twitter], it’s filled with misinformation- blue check marks are bot accounts to farm likes for cash, account doesn’t seem like genuine, would be paid bot account. [FG, 17yo, vaccinated] The TikTok video is less believable because anyone can dance with scrubs on. I have no idea if she's a real doctor. [IDI, 14yo, vaccinated]

^a^ELM: Elaboration Likelihood Model.

^a^HPV: Human papillomavirus.

### Knowledge, Attitudes, and Beliefs About HPV and HPV Vaccine

Adolescents’ knowledge, attitudes, and beliefs about HPV and the vaccine varied notably by age and vaccination status. All vaccinated adolescents aged 16-17 years (n=5) demonstrated the most comprehensive understanding, often recognizing that HPV affects both males and females and that the vaccine is recommended for all adolescents regardless of gender. In contrast, two-thirds of unvaccinated adolescents aged 14-15 years (2/3) held the misconception that the HPV vaccine is only for girls, and several reported hearing about it for the first time during the study.

All vaccinated adolescents aged 14-15 years (n=6) were aware that they had received the vaccine but lacked clarity about what HPV is, how it is transmitted, and what symptoms it causes. They described vaccination as something that happened during a routine physician visit, without detailed explanation. Approximately 80% of the vaccinated and unvaccinated adolescents aged 16-17 years (7/9) generally knew that HPV is a sexually transmitted infection and could name potential consequences of infection, including warts and cancer. These older participants had also heard about methods of prevention beyond vaccination, such as using protection during sex. Overall, knowledge was limited among younger adolescents, particularly those who were unvaccinated, whereas older participants—regardless of vaccination status—were more informed and engaged in thinking critically about HPV and its risks.

### Barriers and Motivators to HPV Vaccine Uptake

Among all vaccinated adolescent males, parental influence—notably that of their mothers—was a consistent motivator for HPV vaccine uptake. A total of 80% (4/5) of the vaccinated adolescents aged 16-17 years described shared decision-making with their parents before receiving the vaccine, during which they discussed the benefits of the vaccine, asked questions, and ultimately agreed to get vaccinated. Some recalled learning about HPV from their parents or pediatricians, reinforcing their confidence in the decision. In contrast, 80% (5/6) of vaccinated adolescents aged 14-15 years did not have conversations with their parents or physicians before receiving the vaccine. Despite limited involvement in the decision-making process, most expressed trust in their parents’ choices regarding their health.

Unvaccinated adolescents identified several barriers to vaccination. Across age groups, concerns about adverse effects and parental skepticism were prominent. All unvaccinated adolescents aged 14-15 years (n=3) believed that vaccination was not necessary because they were not engaging in sexual activity through which HPV could be contracted, reflecting a perception that the vaccine is only needed once someone becomes sexually active. Many felt that “if you’re safe, you will be fine.” All unvaccinated adolescents aged 16-17 years (n=4) were more cautious about vaccine uptake because of adverse effects commonly associated with vaccine, which was often influenced by parental concerns. Despite hesitancy, many unvaccinated adolescents from both age groups expressed interest in learning more and emphasized that they would want to conduct their own research before deciding whether to be vaccinated.

### Social Media Use

#### Overview

Across vaccination status and age groups, adolescent males noted that they were on social media daily, primarily for entertainment and to chat with friends. Instagram was reported as the most popular platform for everyday social media use. TikTok, YouTube, and X (formerly known as Twitter) were also noted as platforms used across age groups. Among older adolescents aged 16-17 years, platforms such as Snapchat and Discord were especially prevalent and were often used for direct messaging, gaming-related conversations, and school-related group chats. Reddit was more commonly mentioned by younger adolescents aged 14-15 years, who used it to follow specific interests and read anonymous discussions. While social media was part of everyday life, few participants reported using it intentionally for health information.

#### Social Media Use for Health Information

Across vaccination status and age group, adolescent males indicated that they do not seek health information on social media because of concerns regarding the reliability of information circulating online. When asked what types of information they would want HPV social media content to address, many noted that they would like to understand the risks of contracting HPV, how the vaccine would mitigate those risks, and where to obtain the vaccine. Similarly, many adolescents noted that they wanted to understand the risks associated with vaccination and how vaccination might affect their future health if they were to receive the vaccine (eg, fertility issues in the future).

### Trust and Believability of Vaccine Messages on Social Media

Across all age groups and vaccination statuses, adolescent males noted that social media is not their first source for accurate, reliable health information. Most participants said they would not trust HPV vaccine content on social media, with some saying they would trust it if the content was from a trustworthy source. A total of 67% (12/18) of participants said they would do more research to understand which social media posts are accurate and trustworthy and would seek multiple sources for health information given the spread of misinformation on social media.

When asked about trusted sources, older male adolescents noted that they would turn to their parents or personal physician for reliable and accurate information about HPV. Approximately 60% (3/5) of vaccinated older adolescents also said they trust health information from organizations such as the World Health Organization, Mayo Clinic, or another major hospital. About 40% (2/5) of older adolescents also said they would trust content that their peers, classmates, friends, or family members shared on social media.

When shown the 4 social media narratives, adolescent males, regardless of age, noted that the Maryland Department of Health post was trustworthy because it provided facts and was from a reliable source. There were mixed results, leaning to not trustworthy, about the InfoWars post because the source cited its own article, and many adolescents did not know the source. ​All adolescents noted that the TikTok narrative was not believable because messaging with singing and dancing was deemed unserious. A little over 80% (15/18) of participants noted that the narrative from Robert F Kennedy Jr would warrant a conversation with their physician or parent about the adverse effects of the HPV vaccine. However, two-thirds of unvaccinated adolescents aged 14-15 years (n=3), believed that narrative because they knew Robert F Kennedy Jr and had heard about risks associated with vaccination.

Participants found that the persuasiveness of a message was influenced by 3 key factors: content format, the substance of the message, and the source’s credibility. Most participants found the dancing doctor video on TikTok to be “unprofessional,” saying that it does not convey the seriousness of the issue and the content did not seem reliable as presented. On the other hand, the images from the Robert F Kennedy Jr post made it compelling through emotional appeal. Unvaccinated adolescents aged 14-15 years were more likely to believe social media messaging that included personal anecdotes, whereas the adolescents aged 16-17 years were more persuaded by statistics and facts.

Older adolescents also emphasized the importance of examining the account that posted the content. Source credibility emerged as a salient factor for adolescents aged 16-17 years, highlighting that respondents were more likely to evaluate the reliability and validity of health information based on the authority, expertise, and affiliations of the poster. For example, the Maryland Department of Health post was widely considered credible because of its factual tone and clear institutional affiliation. The majority of respondents had heard of the Maryland Department of Health and found that this source would be credible for health information. In contrast, the InfoWars post was largely dismissed, with participants citing unfamiliarity with the source and concerns about its legitimacy.

Some participants said that the platform on which the narrative was posted impacted believability, suggesting that Instagram and Facebook are more trustworthy than TikTok and X. Participants also noted that other post characteristics, such as the date of the post and engagement metrics (eg, likes and reposts), impacted believability.

## Discussion

### Overview

This study underscores the significant role of social media in shaping adolescent males’ knowledge and attitudes toward HPV vaccination. Consistent with the study aims, we found that adolescent males’ engagement with HPV vaccine messaging on social media varies by age and vaccination status. Notably, unvaccinated and younger adolescent males exhibited lower awareness of HPV and the vaccine, rendering them more susceptible to misinformation prevalent on social media platforms. This aligns with findings from a systematic review indicating that adolescents often distrust online health information but continue to use it, using various heuristics to assess credibility, such as the source’s perceived trustworthiness and the content’s presentation [39]. The preference for information from familiar or relatable sources, including personal physicians and mothers, highlights the need for targeted health communication strategies that resonate with this demographic.

Furthermore, our research revealed that the believability of social media messages was predominantly influenced by the source rather than the content or format of the post. Older adolescent males demonstrated a higher propensity to trust messages from credible sources, such as health care providers and reputable organizations, and were more likely to engage with authoritative websites such as the CDC and Mayo Clinic for health information. Conversely, younger, unvaccinated males were more inclined to believe anecdotal messages featuring personal stories, which can be compelling but may not always present accurate or comprehensive information. This finding is consistent with studies showing that adolescents’ trust in health information on social media is complex and influenced by factors such as platform trustworthiness, user identity, and content credibility [21]. Additionally, while parents and personal physicians were universally trusted sources of information for all adolescent males, social media posts featuring unrecognizable physicians were found to be unpersuasive, underscoring the importance of source credibility in health communication. These insights contribute to the growing body of research on the influence of social media on health-related decision-making and highlight the necessity for public health interventions to navigate the evolving digital landscape effectively.

### Alignment With ELM

Our findings align with ELM, which posits that individuals process persuasive messages through either the central or peripheral route depending on motivation and ability to engage with the content. Older adolescent males aged 16-17 years, especially those vaccinated, appeared to engage more often through the central route, processing information thoughtfully, evaluating arguments, and preferring content from trusted, authoritative sources. Their critical evaluation of message credibility, preference for fact-based content, and desire to consult external sources (such as physicians or reputable websites) are all hallmarks of central-route processing.

In contrast, younger adolescents were more likely to engage through the peripheral route, relying on emotional cues, personal anecdotes, and familiarity rather than systematic analysis. They tended to be more receptive to personal narratives from public figures or peers, relying on heuristic processing. These distinctions underscore the importance of tailoring message design not only by age but also by presumed level of cognitive engagement, which ELM helps conceptualize effectively. This theoretical grounding offers a valuable lens for public health practitioners aiming to design persuasive health messages based on audience readiness, message processing style, source credibility, and story-telling content.

### Strengths and Limitations

This study has many strengths. First the research team’s ability to engage directly with adolescent males through a nationally representative probability panel was a major strength. This group is often a difficult-to-reach population in health communication research; however, we were able to explore their nuanced perspectives on vaccine messaging and social media use through innovative recruitment efforts. Second, the use of a nationally representative, probability‑based recruitment panel enhances the generalizability of findings to US adolescent males by capturing perspectives across diverse geographic regions and vaccination statuses rather than relying on convenience samples. Lastly, by using real-world examples of social media narratives, we captured insights grounded in participants’ lived experiences, increasing the validity of our findings.

However, this study is not without limitations. First, despite using a nationally representative panel for recruitment, the final participant pool was relatively homogenous in terms of race/ethnicity and socioeconomic background, which may limit generalizability. Our sample was predominantly White and non‑Hispanic. This homogeneity in our adolescent sample may have resulted in greater trust in clinicians and well‑known public health agencies than would be observed among individuals with differing racial/ethnic and socioeconomic backgrounds. Similarly, participants described low reliance on social media for health decisions and a preference for “official” sources, noting that their information-seeking behaviors may not be the norm. Evidence suggests that Black, Hispanic/Latine, immigrant, and economically disadvantaged communities can experience higher medical mistrust toward government, pharmaceutical companies, and, at times health care systems, rooted in historical and ongoing inequities; such mistrust is associated with lower HPV vaccine uptake and different messenger preferences (eg, family networks, faith leaders, and community‑based organizations).

Second, while the original study design emphasized focus groups to encourage peer discussion, recruitment challenges, particularly among adolescents aged 14-15 years, necessitated a pivot to individual interviews. As a result, we were not able to fully explore group dynamics or peer influence, which may have provided additional regarding social norms and vaccine attitudes. It is important to note that this transition may have limited the ability to capture peer-to-peer influences that are particularly salient during adolescence. Group discussions may have revealed additional insights into how social norms, shared beliefs, and peer reinforcement shape perceptions of vaccine-related content. While individual interviews allowed for a safe space to discuss this sensitive topic and were effective in eliciting personal reasoning and decision-making processes, the absence of group interaction may have obscured how peer discussions and perceived norms influence older adolescents’ confidence in, or resistance to, vaccine messaging.

Lastly, although the study leveraged the AmeriSpeak parent–teen panel to reduce recruitment bias, reliance on parental consent may still introduce some degree of selection bias. Parents and adolescents were consented into the AmeriSpeak panel before learning about individual study topics, which helps mitigate overrepresentation of parents with particularly strong provaccine views. Additionally, both parents and adolescents completed vaccine‑related screener items that allowed us to capture a range of vaccine attitudes.

Future research should explore alternative strategies to better engage younger adolescents, including school-based recruitment partnerships, collaboration with pediatric practices, and integration of research activities into youth-centered digital environments such as moderated online communities or gamified platforms. In addition, incorporating parent-adolescent dyadic designs may also enhance participation while capturing the shared decision-making dynamics that are central to HPV vaccination at this age.

### Implications for Future Communication Efforts

The distinct differences in how younger and older adolescent males engage with and interpret vaccine messaging on social media suggest that a one-size-fits-all approach would be ineffective. Instead, interventions should be tailored by age group, vaccination status, and other key factors such as health information seeking behaviors and use of digital tools (social media, generative artificial intelligence [AI], etc). For younger adolescents, messaging should likely target both the adolescent and their parents, given the significant influence of parental decision-making in this age group and the limited health literacy among adolescents aged 14-15 years. Messaging for this audience should use emotionally engaging content that includes personal stories and encouragement for younger adolescents to have conversations with their parents and physicians when engaging with social media health information posts that need further context. A testable hypothesis for future research while using the principles of ELM, could examine whether, among younger adolescents with lower baseline knowledge, brief narrative posts paired with visible peripheral cues of credibility (eg, verified badges and institutional logos) would increase perceived relevance and discussion with parents more than statistics‑heavy posts.

Messaging for older adolescents should be grounded in facts and delivered by highly credible sources, such as pediatricians or well-known public health agencies. Delivery platforms that are familiar and accessible to both youth and parents, such as Instagram and Facebook, may be more effective than TikTok or X. For older adolescents, who are increasingly autonomous in their health decisions, messaging should include more fact-based content with direct appeals to logic, data, and the consequences and risks of not vaccinating. This audience showed a higher likelihood of engaging in their own research with reputable websites or posts from verified sources, indicating a preference for content that satisfies a need for accurate, actionable information. A testable hypothesis for future research, while using the principles of ELM, could examine whether older adolescents with higher motivation who receive fact-forward messages from identifiable health care professionals would demonstrate higher message credibility and intent to vaccinate compared with entertainment-style narratives without evidence-based content. These findings suggest future HPV vaccine campaigns should develop parallel message strategies for different developmental stages: one focusing on emotional resonance and parental influence for younger adolescents and the other emphasizing autonomy, scientific rationale, and source credibility for older adolescents.

### Conclusion

#### Summary of Key Findings

This study sought to explore how adolescent males engage with and interpret HPV vaccine-related messaging on social media, with a focus on differences by age and vaccination status. Findings indicate that younger and unvaccinated adolescents demonstrate lower HPV awareness and greater susceptibility to misinformation, whereas older adolescents are more likely to engage critically with health information and seek content from credible, authoritative sources. Social media plays a central yet complex role in shaping perceptions, with message credibility often hinging on the source rather than the content or format. Trust in personal physicians and parents remains strong, and different age groups exhibit distinct preferences for how and from whom they receive health information.

#### Public Health Implications and Practical Applications

These findings have important implications for both HPV vaccination efforts and the broader field of adolescent health communication. Public health practitioners should consider age-segmented strategies tailored to developmental stages and align message framing, emotional tone, and source credibility with adolescents’ decision-making autonomy when designing campaigns. For younger adolescents, practical applications include developing social media content that encourages parent-adolescent conversations and uses emotionally resonant narratives while clearly directing families to trusted health care providers for follow-up information. For older adolescents, campaigns may be more effective when they emphasize factual content, transparent risk-benefit information, and messages delivered by identifiable, reputable health professionals or organizations.

Health care providers and public health agencies can also apply these findings by strategically integrating social media into vaccine counseling, using trusted platforms to reinforce in-clinic conversations rather than replace them. Training clinicians and public health practitioners to recognize how youth interpret social media narratives may enhance their ability to address misinformation and tailor recommendations during clinical encounters. Additionally, vaccine campaigns should prioritize platforms familiar to both adolescents and parents to maximize reach and credibility.

#### Future Research Considerations

As the digital ecosystem continues to evolve rapidly, it is also important to explore emerging technologies, such as generative AI, as potential tools for delivering reliable, factual vaccine content to youth. Understanding how adolescent males interact with AI-generated information, alongside traditional social media, will be critical for designing future interventions that keep pace with their digital footprint. Studies incorporating parent-adolescent dyads, peer-based interactions, and more diverse populations are also needed to better understand shared decision-making processes and social influences on HPV vaccine uptake. Longitudinal designs may further clarify how adolescents’ message processing and trust in digital health information change over time.

## Data Availability

Data sharing is not applicable to this article as no data sets were generated or analyzed during this study.

## Appendix

Multimedia Appendix 1 Moderator guide for exploring the role of social media narratives and hpv vaccine confidence among adolescent males.

Multimedia Appendix 2 Social media posts.

## Abbreviations

AI: artificial intelligence
CDC: Centers for Disease Control and Prevention
ELM: Elaboration Likelihood Model
HPV: human papillomavirus
IRB: institutional review board
NORC: National Opinion Research Center
