# Supplementary material for: The Influence of Social Media Messaging on Human Papillomavirus Vaccine Attitudes and Confidence Among Adolescent Males: Group Discussion Study
Source: JMIR Cancer. 2026 Jun 3;12:e82210. doi: 10.2196/82210 (PMC13276466; doi:10.2196/82210)
Supplement: Multimedia Appendix 1 [file cancer_v12i1e82210_app1.docx]

**Multimedia Appendix B.**

***MODERATOR GUIDE FOR EXPLORING THE ROLE OF SOCIAL MEDIA NARRATIVES and HPV VACCINE CONFIDENCE AMONG ADOLESCENT MALES***

**Introductions, Ground Rules, Explanation of Focus Group Process, Consent (5 min)**

**Introduction:**

Good morning/afternoon! My name is [*Name*] and I will be facilitating the discussion this afternoon. This is [*Name 2*] and he/she will be taking notes and helping me. Thank you for joining our discussion today. On behalf of Merck and in collaboration with Thomas Jefferson University, NORC at the University of Chicago is conducting this work to learn about the role of social media in young adults’ HPV vaccine knowledge and attitudes.

Our discussion will take about 60 minutes. Our goal today is to learn about your knowledge about HPV and the HPV vaccine, the role of social media in your health decision-making, and the believability of social media messages. We encourage you to share your open and honest thoughts and feelings. There are no wrong answers, and your opinions are incredibly valuable to use.

**Ground Rules:**

I would first like to go over some ground rules for today’s discussion.

- The most important ground rule is that only one person speaks at a time, so that we can hear each other clearly. This will also help us take good notes.
- Please speak clearly and loudly enough for everyone to hear you.
- You can raise your hand to speak or speak up after the other person has finished speaking.
- There are no right or wrong answers.
- You do not have to agree with the views of other people in the group. In fact, if you disagree we hope you will share your perspective so we can understand the full range of views in the group.
- You do not have to speak in any particular order.
- You should feel free to speak directly to each other – this is a group conversation, so you don’t have to address all your comments to me.
- To make this a more lively conversation, please feel free to turn on your camera!
- There are many of you in the group and it is important that we hear from all of you. Some of you may be more comfortable speaking than others, so at times I may ask more talkative people to let others speak first or invite a quieter person to share their thoughts. If you are speaking, please be mindful of the language you use.
- It’s also important that everyone is able to pay attention to the discussion with minimal distractions. Please take the call from a quiet space and turn off your phone or other electronic devices that may distract from the discussion.

**Informed Consent**

Before we begin with this discussion, I would like to go over the informed consent.

We will keep everything you say in this discussion private. When we share what we’ve learned from this group, we will not include any names or other identifying information. We also ask you to maintain each other’s privacy and not discuss with others what was shared here today.

As I mentioned, this discussion will last about 60 minutes. If at any point you do not want to continue participating in this discussion, you are free to leave the group. You can also skip any of the questions if you’d rather not answer.

We would like to audio record this discussion in order to ensure our notes are accurate. All notes and the recording will be kept safely and securely. We will delete the recording at the end of the project. Is everyone okay with recording this conversation? [*Confirm that all participants consent & start recording*].

As I noted earlier, today’s discussion will help us understand the role of social media in impacting HPV vaccine confidence among adolescent males. There is no known physical risk to you from being in this project. To thank you for your time, everyone who completes the focus group will receive 50,000 AmeriPoints, worth the equivalent of $50. Some of the people working on the project will be observing the focus group to take notes. If you have any questions, you may contact Neha Trivedi, Project Director, at (202) 738-9865‬ ‬or Trivedi-Neha@norc.org.

Do you agree to participate?

- Yes
- No

*[If respondent(s) do not agree to participate and answers “No”: Thank them, and ask them to disconnect from the line.]*

Does anyone have any questions before we begin? [*Answer any questions*]

1. **Introductions (5 minutes)**

Before we dive in, I’d like for us to get to know one another a little.

1. Please tell us your first name (or a nickname, or even a fake name), the city where you live, and a superpower you would like to have.
2. **Knowledge about HPV and the HPV Vaccine (10 minutes)**

That was fun! Now, I’d like to move us onto the main discussion.

1. To get started, I’d love to hear from each of you, have you heard of HPV or human papillomavirus?
   1. **For those who have heard about HPV**, what do you know about it?
   2. Do you know how someone gets HPV?
   3. How concerned are you about getting HPV at some point in your life?
   4. What health problems can it cause?
   5. How concerned are you about the health problems HPV can cause?
2. Have you heard about a vaccine for HPV? If so, what have you heard?
   1. Where did you hear about it?
   2. Did you know it was available for boys?
3. **Attitudes, Beliefs, Barriers, and Motivators towards HPV Vaccination (10 minutes)**
4. **FOR VACCINATED GROUP/PARTICIPANTS:** Do you remember getting the HPV vaccine?
   1. How did you decide to get the HPV vaccine? Did someone help you make the decision?
      1. Was it you, your parent(s), or your doctor?
      2. Did you and your parent(s) talk about the decision for you to get the HPV vaccine? If so, what was that conversation like?
   2. If you become a parent in the future, do you think you'll get the HPV vaccine for your son or daughter? Why or why not?
5. **FOR UNVACCINATED GROUP/PARTICIPANTS:** Do you know why you haven't gotten the HPV vaccine?
   1. Why did you decide to not get the HPV vaccine? Was there anyone who was part of this decision?
   2. Do you, personally, have any concerns about getting the HPV vaccine?
   3. Are there things you would want to know before deciding to get the vaccine?
6. **Social Media Platform Use, Health Information Seeking, and Trust/Distrust in Messages on Social Media (15 minutes)**

Thanks so much for sharing all that! Now I’d like to hear from everyone about your social media use, how you get your health information, generally, and who you trust for health information.

1. In a given day, how do you use social media?
2. Do you ever look for health information on social media? If so, what are you looking for?
   1. What platforms do you look for health information on?
      1. TikTok, YouTube, Twitch, Reddit, Snapchat, Instagram
   2. **If not currently looking for health information on social media**: What platform would you go to if you wanted to learn something or had a question about your health?
   3. Why do you prefer those channels?
   4. Who do you like to hear from about health information? Who will you listen to or take advice from?
3. Have you ever seen posts about the HPV vaccine on these platforms?
   1. **For those who have:**
      1. What did you see?
      2. Did you trust it? What made it trustworthy?
      3. Did it influence your knowledge, attitudes, or behaviors related to the HPV vaccine?
   2. **For those who haven’t:**
      1. How would you react if you saw HPV vaccine content on social media?
      2. Would you trust HPV vaccine content on social media?
4. What would you like HPV vaccine content on social media to be about?
   1. For example, the risk of HPV, how to get the vaccine, who is eligible, etc.
5. What type of person or messenger would you trust for information about the HPV vaccine on social media?
   1. For example, influencer, peer, provider, health department?
6. **Testing HPV Social Media Narratives (15 minutes)**

Thanks so much for those insights! Now I’d like to show you a few social media posts about the HPV vaccine and get your thoughts.

[Screenshare social media narratives/graphics/messages here – show one post at a time and ask Q11-14 for each post, then after they’ve seen all posts ask Q15-17.]

1. What are your first impressions of this post?
2. Do you believe this message? What about this message is believable?
3. Does seeing this message make you think differently about the HPV vaccine? If so, in what ways?
4. What did you think of the platform on which it was shared?
   1. What did you think of the poster?
5. What aspects of the messages are most persuasive? Which aspects are least persuasive or compelling?
   1. Prompt: the content (statistics, anecdotal stories), the type of content (video, images, text), source/poster, platform, caption length/tone, hashtags
6. Which of these messages would make you more likely to get the HPV vaccine? Which of these messages would make you less likely to get the HPV vaccine?
7. Would any of these messages prompt conversations about the HPV vaccine with your parents and/or doctor? What about that post would prompt those conversations?

**Closing**:

That’s it! Thanks so much for your time. If this conversation has prompted questions about HPV vaccine and you’d like to learn more, please visit: https://www.cdc.gov/hpv/vaccines/index.html

Before we wrap up today…

Do you have any additional feedback that you want to share with me today?

Do you have any questions for me at this time?

Thank you for your time. We appreciate you all for sharing your thoughts with us. Your 50,000 AmeriPoints (worth $50) will be uploaded straight into your account and will show in your portal within 10 business days.
