# Supplementary material for: The Influence of Social Media Messaging on Human Papillomavirus Vaccine Attitudes and Confidence Among Adolescent Males: Group Discussion Study
Source: JMIR Cancer. 2026 Jun 3;12:e82210. doi: 10.2196/82210 (PMC13276466; doi:10.2196/82210)

**Multimedia Appendix A.**

Narrative #1: X (formerly known as Twitter)


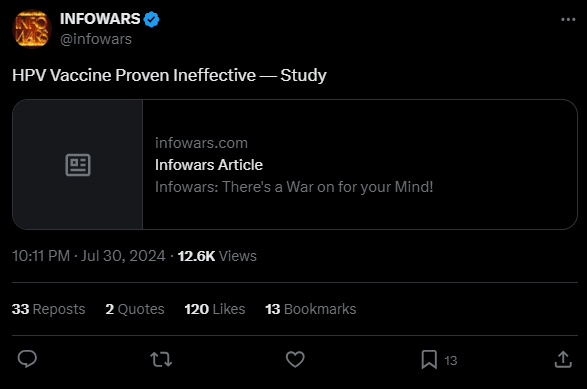


Narrative #2: Instagram


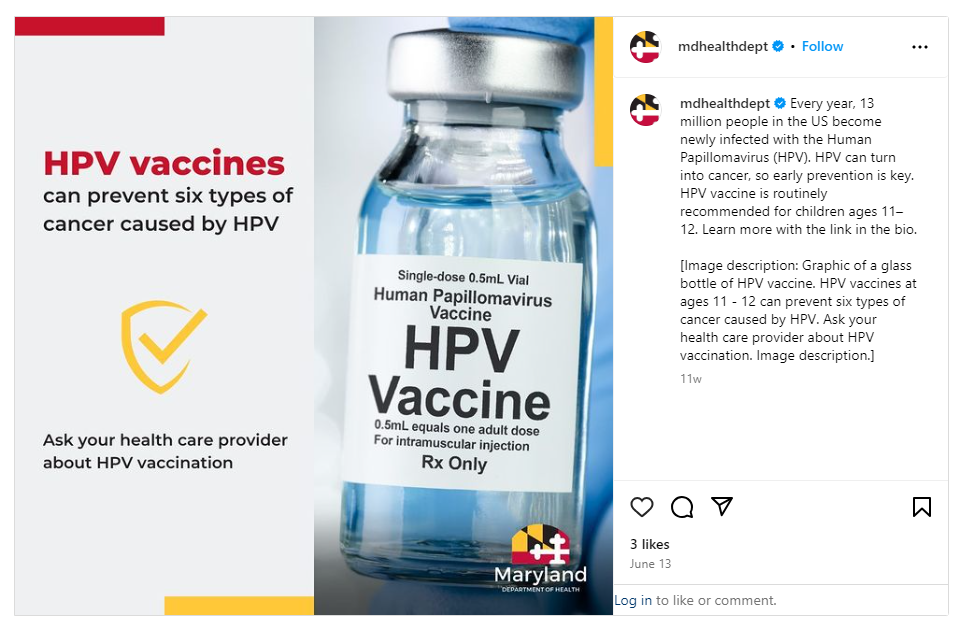


Narrative #3: TikTok


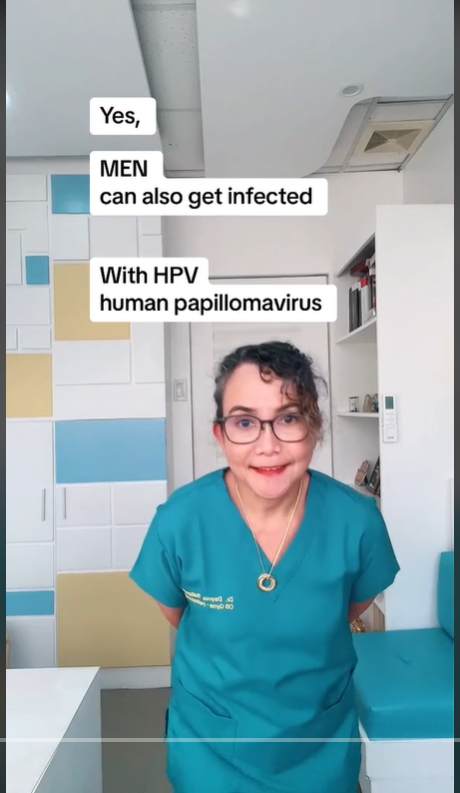


Narrative #4: Facebook


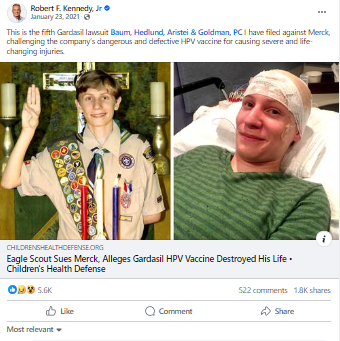

Supplement: Multimedia Appendix 2 [file cancer_v12i1e82210_app2.docx]
